# The Relationship between Attitudes toward Suicide and Family History of Suicide in Nagano Prefecture, Japan

**DOI:** 10.3390/ijerph13060623

**Published:** 2016-06-22

**Authors:** Teruomi Tsukahara, Hiroaki Arai, Tomoko Kamijo, Yoshikiyo Kobayashi, Shinsuke Washizuka, Heihachiro Arito, Tetsuo Nomiyama

**Affiliations:** 1 Department of Preventive Medicine and Public Health, School of Medicine, Shinshu University, 3-1-1 Asahi, Matsumoto, Nagano 390-8621, Japan; h_arai@shinshu-u.ac.jp (H.A.); kamijoh@shinshu-u.ac.jp (T.K.); nomiyama@shinshu-u.ac.jp (T.N.); 2 Department of Occupational Medicine, School of Medicine, Shinshu University, 3-1-1 Asahi, Matsumoto, Nagano 390-8621, Japan; harito@fancy.ocn.ne.jp; 3 Nagano Prefecture Saku Health and Welfare Office, 65-1 Atobe, Saku, Nagano 385-8533, Japan; kobayashi-yoshikiyo@pref.nagano.lg.jp; 4 Department of Psychiatry, School of Medicine, Shinshu University, 3-1-1 Asahi, Matsumoto, Nagano 390-8621, Japan; swashi@shinshu-u.ac.jp

**Keywords:** suicide, attitude toward suicide, family history of suicide, postvention

## Abstract

Certain attitudes toward suicide may be a risk factor for suicide among the bereaved. To explore this possibility, we examined the relationship between attitudes toward suicide and family history of suicide. We focused on two specific attitudes indicating resignation in a survey: #1 “When a person chooses to die by suicide, the suicide is inevitable” (*i.e.*, inevitability belief); and #2 “A suicide cannot be stopped by any person, because suicide is unpreventable” (*i.e.*, unpreventable belief). The data of 5117 fully completed questionnaires were analyzed. Logistic regression analysis revealed that the two attitudes of resignation were significantly associated with a family history of suicide. The adjusted odds ratio for #1 was 1.39 (95% CI, 1.07–1.79) for individuals having experienced suicide by a family member or relative, while that for #2 was 1.57 (95% CI, 1.27–1.95) for experiencing a suicide by a family member or relative and 1.25 (95% CI, 1.05–1.49) for experiencing a suicide by a friend, business associate, partner or other. These two attitudes of resignation toward suicide were significantly associated with a family history of suicide. These attitudes might increase suicide risk among the bereaved.

## 1. Introduction

According to the World Health Organization (WHO), approximately 800,000 individuals worldwide die annually by suicide, making the prevention of suicide an important public health issue [1]. In the WHO’s 2014 report, Japan had the fifth-highest rate of suicide in the world [1]. In a 2014 White Paper, Japan’s Cabinet Office noted 27,283 suicides and a crude suicide rate of 21.4 suicides per 100,000 residents [2]. The annual number of suicide deaths in Japan exceeded 30,000 during the 14-year period from 1998 to 2011, and then slowly declined to approximately 27,000 [2]. In Nagano Prefecture where we conducted this survey, the yearly number of suicide deaths in recent years was approx. 500, representing a crude suicide rate of 20.1 per 100,000 residents, which is average among the 47 prefectures of Japan [3].

The close relationship between the deceased and the bereaved is associated with negative health and social outcomes for the bereaved. Consequently, the risk of suicide among the bereaved is elevated [4]. The bereaved, especially the families and relatives of someone who has died by suicide, often experience stigma linked to the event, such as feeling ashamed, increased rejection, and may receive inadequate social support [4,5,6,7,8,9]. In addition, the bereaved tend to be subjected to a very complicated form of bereavement with a deep grief reaction [10]. This grief [5,9] and stigma [7] hinder the bereaved’s efforts to seek help and may inhibit other people from offering support, thereby raising the threshold for death by suicide among the bereaved [1]. We reported in our companion paper [11] that suicidal ideation was significantly associated with the feeling ashamed for seeking help when distressed, indicating a possible decrease in the threshold for suicidal behaviors, such as suicidal ideation, among the bereaved.

Therapeutic postvention targets the bereaved. The postvention process aims to not only offer timely support to the bereaved, but also prevent “suicide pacts” among the bereaved by eliminating the risk factors involved in this event. Especially, inappropriate attitudes toward suicide are important risk factors targeted by postvention strategies [1]. In this paper we defined the attitude which is a favorable or unfavorable evaluative reaction toward something or someone, exhibited in one’s beliefs, feelings, or intended behavior [12].

In two nationwide surveys [13,14], Japan’s Cabinet Office examined six attitudes regarding suicide among 2017 participants living in various regions of Japan. Among the six attitudes toward suicide, two, *i.e.*, “When a person chooses to die by suicide, the suicide is inevitable”. and “A suicide cannot be stopped by any person, because suicide is unpreventable”. were notably different from the other four attitudes with respect to the rates of people’s agreement with the attitude. Kageyama [15], using the same six attitudes toward suicide used in the Japan Cabinet Office’s surveys, reported that these two inappropriate attitudes toward suicide (which regard suicide as an inevitable and unpreventable event), are significantly associated with living in high suicide-mortality areas [13,14]. It is thought that bereaved individuals may take on a resigned attitude toward suicide as an inevitable and unpreventable event. These two attitudes toward suicide, *i.e.*, an attitudes of resignation toward suicide, might be influenced by a familial aggregation of suicide as suggested by Joiner and Van Orden [16,17], and they might serve as one of the factors that could be used to evaluate the suicide risk among the bereaved. These two attitudes were emphasized because individuals who have “attitudes of resignation” might be unable to make the distressed person to access a healthcare system. Therefore we emphasized the attitude of resignation toward suicide in this study.

Effective suicide-prevention postvention support in the healthcare system is urgently needed for the bereaved in order to lower their suicide risk. For example, the WHO [18] recommends the formation of self-help support groups to provide opportunities for the bereaved to counter their depression, anxiety and despair. The present exploratory study examined the relationships between suicide in an individual’s life and his or her attitude toward suicide, focusing on residents of Nagano Prefecture, with an emphasis on the relationship between the two above-described attitudes toward suicide and the existence of a family history of suicide.

## 2. Materials and Methods

### 2.1. Study Design and Participants

Details of the study design have been published in our companion paper [11]. The anonymous questionnaire that we used was originally designed to examine mental health problems linked to suicide among residents of Nagano Prefecture. The Nagano prefectural government asked all 77 of its municipalities to participate in the survey, and 14 municipalities (18.2%) agreed to participate. Study participants of both sexes over 20 years of age were randomly selected from the 14 municipalities according to the age composition rate in each municipality. In principle, the municipal officials in charge of the survey sent the questionnaire to candidate participants by mail together with a briefing paper informing them of the objective and methods of the survey, the publication of the results, the option to decline to participate, and an explanation of informed consent (which was to be provided orally). The participants sent the questionnaires back by mail to the local officials in an enclosed envelope. We considered that informed consent had been given when a participant returned the questionnaire. Of the 11,100 residents who were sent the questionnaire, a total of 7394 participants returned the questionnaire, and the data of the 5117 participants who responded properly to all the questionnaire items were subjected to the present analyses.

### 2.2. Questionnaire Survey

We analyzed the questionnaire items including suicide that had occurred among people that the participant knew and the participant’s attitudes toward suicide, his or her Kessler 6 (K6) score, gender, age and occupational status. Occupational status was classified into the seven categories shown in Table 1. Depressive symptoms were evaluated with the Japanese version of Kessler’s K6 scale [19]. The K6 mental health scale is a six-item self-report asking how frequently the participant had experienced symptoms of psychological distress during the past 30 days.

We classified the participants into two groups: those with depressive symptoms (a total K6 score of ≥ 5) and those without depressive symptoms (a total K6 score of 0–4) according to the recommended cutoff point [19,20,21]. Our questionnaire assessed the participant’s experience with a suicide in his or her life by asking the question: “Has someone you know died by suicide?”. In the case of “yes”, the participant was further asked about who died by suicide: a member of his or her immediate family, another relative, a friend, a business associate, a partner and/or others. It was possible to check off more than one person. We classified the suicides into three different groups: members of the participant’s immediate family (*i.e.*, parent, child, spouse, or sibling) and other relatives (*i.e.*, grandparent, cousin, uncle or aunt), abbreviated as “FAM”; friends, business associates, partners and others, abbreviated as “FRND” and no suicide in the participant’s life as “None”. In this study, we defined “family history of suicide” as a suicide by an immediate family member or other relative. When they had a both history of suicide, we classified that they had a history of suicide of family. Because we assumed that the familial close relationship might affect their attitudes toward suicide.

The participants were asked to read the following six attitudes toward suicide, which are quoted from the Cabinet Office’s nationwide surveys on suicide [13,14]:

#1: “When a person chooses to die by suicide, the suicide is inevitable”.
#2: “A suicide cannot be stopped by any person, because suicide is unpreventable”.
#3: “Something good will happen if a person chooses to live rather than die by suicide”.
#4: “The decision whether to live or to die should be entrusted to the other individual’s personal judgment”.
#5: “The idea of suicide pact should be abandoned when there are one or more children to consider”.
#6: “Individuals who have died by suicide must have experienced an extremely difficult time”.

We interpreted attitude #1 and attitude #2 as “inevitable belief” and “unpreventable belief” respectively. Furthermore, we regarded these two attitudes as “attitudes of resignation” which might hinder the suicide prevention.

The questionnaire asked the participants to answer “agree” “agree a little” “disagree” “disagree a little” or “neither agree nor disagree” for each of the six attitudes toward suicide. We classified these responses into three different groups: “agree and agree a little” as “Agree”, “disagree and disagree a little” as “Disagree” and “neither agree nor disagree” as “NAD”. We assumed that some participants might feel uncomfortable responding to the question about the six attitudes toward suicide, and we thus added the following sentence to the questionnaire: “If you feel uncomfortable, you don’t need to complete this section about the six different attitudes toward suicide”. The percentages and numbers of the participants who did not answer six attitudes was 30.8% (2277/7394).

### 2.3. Ethical Considerations

We obtained informed consent directly from the participants by returning the questionnaire. The municipal health officials were given the authorization to conduct the survey from the head of each municipality. Only the participants who returned the questionnaire were enrolled in the present survey. The basic document relating to oral informed consent from the participants on the basis of the briefing paper was recorded and preserved by the health officials under the auspice of the Committee for Preventive Measures against Suicide organized in the Nagano Prefectural Government. The protocol of the survey including the procedure to obtain the informed consent was approved and implemented by the Committee. This study and protocol including the procedure to obtain the informed consent from the participants were also approved by the Ethics Review Committee of Shinshu University School of Medicine [11]. The ethical approval code from the Ethics Review Committee of Shinshu University School of Medicine for our study is 3147.

### 2.4. Statistical Analysis

We performed a trend analysis with the Mantel-Haenszel test. A multiple logistic regression analysis was performed for the determination of the odds ratio with a 95% confidence interval (95% CI) for associations between the attitudes toward suicide as a dependent variable (which was analyzed as “agree” or “disagree”); the independent variables were the participant’s experience with a suicide [9], gender [2], age [2], occupational status [2], and K6 score [19]. A *p*-value less than 0.05 was considered statistically significant. All analyses were conducted using the Statistical Package for Social Sciences (SPSS) ver. 21.0 by IBM (SPSS Inc., Chicago, IL, USA).

## 3. Results

We examined the attitudes toward suicide, depressive symptoms and sociodemographic characteristics of the 5117 participants with complete responses to all of the questionnaire items with respect to suicide in their lives, as shown in Table 1. The rate of participants experiencing suicide in the FAM was 15.5% ((330 males + 461 females)/5117), and that of the participants experiencing suicide in a FRND was 30.7% ((786 males + 783 females)/5117). The rate of participants who had not experienced suicide in their lives was 53.9% (1268 males + 1489 females)/5117).

The rate of “Agree” responses to attitude “inevitable belief” (“When a person chooses to die by suicide, the suicide is inevitable”) was 9.8%, and that of “Agree” responses to attitude “unpreventable belief” (“Suicide cannot be stopped by any person, because suicide is unpreventable”) was 16.4%. The rate of participants with both the existence of depressive symptoms and the experience of a FAM suicide was 37.5%, and that of participants with both depressive symptoms and a FRND suicide was 39.5%. The rate of participants with depressive symptoms and no experience of a suicide by someone they knew was lower, at 31.7%. The rate of participants responding “Agree” to attitude “inevitable belief” was 12.4% among the participants who had experienced a FAM suicide, 9.8% among those who had experienced a FRND suicide, and 9.0% among those who had not experienced a suicide (*p* for trend = 0.02).

The rate of participants who responded “Agree” to (*i.e.*, agreed with) attitude “unpreventable belief” was 20.9% among the participants who had experienced a FAM suicide, 17.6% among those who had experienced a FRND suicide, and 14.4% among those who had not experienced a suicide (*p* for trend <0.01). The rates of participants who agreed with attitudes “inevitable belief” and “unpreventable belief” were in the order of FAM > FRND > None. As summarized in Table 1, the rates of participants who agreed with attitudes #3 through #6 were different from those who agreed with attitudes “inevitable belief” and “unpreventable belief”.

In particular, the rate of participants who agreed with attitude #5 about suicide pact with a child (“The idea of suicide pact should be abandoned when there are one or more children to consider”) was much smaller (at 5.7%) than the agree rates for the other five attitudes toward suicide, regardless of the presence or absence of a suicide in the participant’s life. We selected attitudes “inevitable belief” and “unpreventable belief” for a further logistic regression analysis, since we felt that these two attitudes represent a somewhat resigned attitude toward suicide after experiencing a suicide in one’s life, unlike the other four attitudes (as suggested by Kageyama [15]).

Table 2 summarizes the relationships between attitudes “inevitable belief” and “unpreventable belief” toward suicide along with having had a person in one’s life died by suicide, depressive symptoms and some sociodemographic characteristics. The adjusted odds ratio for attitude “inevitable belief” was 1.39 (95% CI, 1.07–1.79) among the participants who had experienced a FAM suicide, and 1.00 (95% CI, 0.80–1.24) among the participants who had experienced a FRND suicide, in comparison with those who had not experienced a suicide. The adjusted odds ratio of attitude “inevitable belief” was 2.13 (95% CI, 1.70–2.67) in the men compared with women, 2.46 (95% CI, 1.48–4.08) among the participants over 70 years old compared with those who were 20–29 years old, and 2.13 (95% CI, 1.75–2.59) among the participants with depressive symptoms compared with those without depressive symptoms. The adjusted odds ratio for attitude “inevitable belief” was 1.38 (95% CI, 1.00–1.89) among the part-time employees, and 2.09 (95% CI, 1.36–3.22) for other compared with the full-time employees. The weight of the significant association between #1 and a family history of suicide, being a male, an elderly and having depressive symptoms was almost twice in odds ratio respectively.

The adjusted odds ratio for attitude “unpreventable belief” was 1.57 (95% CI, 1.27–1.95) among the participants who experienced a FAM suicide, and 1.25 (95% CI, 1.05–1.49) among those who experienced a FRND suicide, compared with those who had not experienced a suicide. The adjusted odds ratio for attitude “unpreventable belief” was 1.44 (95% CI, 1.21–1.72) for men compared with women, 1.46 (95% CI, 1.02–2.09) among the 60–69-year-old participants and 2.34 (95% CI, 1.58–3.47) among the participants over 70 years old compared with the 20–29-year-old participants, and 1.80 (95% CI, 1.53–2.11) among the participants with depressive symptoms compared with those without depressive symptoms. It is noteworthy that the adjusted odds ratio for attitude “unpreventable belief” among the participants who had experienced a FRND suicide was significant (1.25, *p* < 0.01), but the adjusted odds ratio for attitude “inevitable belief” among the participants who had experienced a FRND suicide was not (1.00, *p* = 0.96).

## 4. Discussion

The results of this cross-sectional study demonstrated that two attitudes of resignation toward suicide are significantly associated with a family history of suicide, together with significant associations of these two attitudes with depressive symptoms, men and age over 60 years old. Our questionnaire used the same six attitudes toward suicide as those used in two nationwide Japan Cabinet Office surveys [13,14], and the rates of our Nagano Prefecture participants agreeing with the six attitudes toward suicide revealed by our questionnaire are in essential agreement with those reported in the two Japan Cabinet Office surveys [13,14]. In particular, the rate of participants agreeing with attitude “inevitable belief” (“When a person chooses to die by suicide, the suicide is inevitable”.) was found to be 9.8% in the present study, which is comparable to the rates of 8.6% and 7.7% for the same attitude reported in the Japan Cabinet Office surveys in 2009 [13] and 2011 [14], respectively.

Notably, the rate (12.4%) of the participants who had experienced a suicide by an immediate family member or other relative and also agreed with attitude “inevitable belief” was significantly higher than the rate (9.0%) of the participants who had not experienced a suicide in their lives and agreed with attitude “inevitable belief”.

In addition, the percentage of participants who had experienced a FAM suicide and agreed with attitude “unpreventable belief” (“A suicide cannot be stopped by any person, because suicide is unpreventable”) was 20.9% in the present study, which is higher than the corresponding 16.4% and 11.8% reported in the Japan Cabinet Office surveys in 2009 [13] and 2011 [14], respectively. We also found that the percentage of participants who had experienced a suicide by an immediate family member or other relative and also agreed with attitude “unpreventable belief” was significantly higher (20.9%) than that of the participants who had not experienced a suicide and agreed with attitude “unpreventable belief” (14.4%).

The present finding that the rates of participants who had experienced a FAM suicide and agreed with either attitude “inevitable belief” or “unpreventable belief” toward suicide were high compared with those who had not experienced a suicide may indicate that the former individuals have a resigned view of suicide as an inevitable and unpreventable event. Kageyama [15] reported significant associations of these two attitudes toward suicide with living in high suicide-mortality areas, suggesting that these two attitudes were formed through experiencing suicide in a family member or friend, *etc.* in a community with a high suicide-mortality rate. Kageyama [15] judged these two attitudes toward suicide as being inappropriate attitudes toward suicide. “FAM” is considered to take an agreeable view toward the first and second attitudes in comparison with no agreeable view toward the fifth attitude among all the participants irrespective of the occurrence or non-occurrence of a suicide in a participant’s lifetime.

“The order of FAM > FRND > None for the rates of participants agreeing with attitudes “inevitable belief” and “unpreventable belief” shows that there is clear dependence of these two attitudes with their experience of suicide. The significant association of attitude “unpreventable belief” with the experience of suicide observed not only in FAM but also in FRND suggests that suicide affects a wide range of people in the community, as suggested by Kageyama [15]. In our study, the people who had experienced a FRND suicide tended to accept both attitudes “inevitable belief” and “unpreventable belief”, whereas those who had experienced a FAM suicide tended to accept only attitude “inevitable belief”. We found in our companion report [11] that suicidal ideation was significantly associated with feeling ashamed for seeking help when distressed among residents in Nagano Prefecture of Japan. On the other hand, the rate of participants agreeing with attitude #5 about not suicide pact was much smaller than the agreement rates for the other five attitudes, suggesting that there was no sympathetic attitude among the participants toward suicide pact, regardless of whether they had experienced anyone’s suicide during their lifetimes.

Our findings revealed that the two resigned attitudes toward suicide (“inevitable belief” and “unpreventable belief”) are significantly associated with not only a family history of suicide but also depressive symptoms. This is in agreement with a study by Qin *et al.* [22], who showed that a family history of completed suicide and psychiatric illness significantly and independently increases suicide risk.

The interpersonal theory of suicide described by Joiner and Van Orden *et al.* posits that a family history of suicide predisposes a bereaved individual to acquire the capability for suicide him- or herself, based on both habituation to the physically painful event of his/her family member’s suicide, and a reduced fear of death [17]. In their 2011 population study of 11.4 million individuals in Sweden, Tidemalm *et al.* [23] observed that a familial aggregation of completed suicide is influenced by genetic and shared-environmental (familial) factors. An earlier series of investigations indicated that although a familial aggregation of suicidal behavior is in part genetic, the aggregation may also be transmitted via a complicated chain of inter-related events in a bereavement-disrupted relationship, contributing to the bereaved’s risk of dying by suicide [24,25,26]. Although we did not obtain genetic information from the participants in our survey, any pattern of familial aggregation might influence their attitudes toward suicide. The attitudes “inevitable belief” and “unpreventable belief” toward suicide might cause a barrier to accessing his or her healthcare system’s suicide prevention program. On the other hand, the individuals who had family history of suicide might be vulnerable to suicide in general and have a guilty conscience. The bereaved might have to represent these attitudes through our questionnaire survey.

## 5. Limitations

First, our analysis of the 14 municipalities’ residents was based on the willingness of municipal officials to participate in the survey, not on random sampling, which could have biased our findings. Second, since this epidemiological study was cross-sectional, the causality of the relationships between a family history of suicide and attitudes “inevitable belief” and “unpreventable belief” toward suicide was beyond the scope of the study. Third, we were not able to examine either the time points of suicide for the group of families and relatives, or the familial relationships between the bereaved participants and the suicide decedents, and we were thus not able to identify any pattern of familial aggregation of suicide within the group. The time points of the suicides and the family relationship might have influenced attitudes “inevitable belief” and “unpreventable belief” toward suicide differently. Fourth, the communities that people live in have an association with risk factors, for example, culture, religious, legal, and economic factors [1]. Our findings are some limitations to generalize to another countries because we did not adjust these risk factors.

## 6. Conclusions

The results of this cross-sectional study showed that two attitudes of resignation toward suicide were significantly associated with a family history of suicide, together with significant associations of these two attitudes with depressive symptoms, men and age over 60 years. Therefore, intervention into the two attitudes of resignation toward suicide among the bereaved is of prime importance for the postvention support in healthcare programs for the prevention of suicide.

## Figures and Tables

**Table 1:** Attitudes toward suicide, depressive symptoms as assessed by the K6 scale, and sociodemographic characteristics of 5117 participants who did or did know someone who had died by suicide.

Variable	Category	Suicide in the Participant‘s Life								*p* for Trend
		Total (*n* = 5117)		FAM (*n* = 791)		FRND (*n* = 1569)		None (*n* = 2757)		
		*n*	%	*n*	%	*n*	%	*n*	%	
Sex	Men	2384	46.6	330	41.7	786	50.1	1268	46.0	
	Women	2733	53.4	461	58.3	783	49.9	1489	54.0	
Age group (years)	20–29	491	9.6	35	4.4	112	7.1	344	12.5	
	30–39	837	16.4	124	15.7	288	18.4	425	15.4	
	40–49	930	18.2	152	19.2	323	20.6	455	16.5	
	50–59	1053	20.6	204	25.8	379	24.2	470	17.0	
	60–69	1115	21.8	178	22.5	332	21.2	605	21.9	
	over 70	691	13.5	98	12.4	135	8.6	458	16.6	
Occupational status	Full-time employee	1942	38.0	293	37.0	661	42.1	988	35.8	
	Part-time employee	814	15.9	129	16.3	242	15.4	443	16.1	
	Business owner/freelance professional	740	14.5	114	14.4	266	17.0	360	13.1	
	Housewife/househusband	656	12.8	136	17.2	157	10.0	363	13.2	
	Unemployed	665	13.0	88	11.1	156	9.9	421	15.3	
	Student	83	1.6	3	0.4	12	0.8	68	2.5	
	Other	103	2.0	28	3.5	75	4.8	114	4.1	
K6 score	≥5	1791	35.0	297	37.5	619	39.5	875	31.7	
	<5	3326	65.0	494	62.5	950	60.5	1882	68.3	
#1: “When a person chooses to die by suicide, the suicide is inevitable”.	Agree	500	9.8	98	12.4	153	9.8	249	9.0	0.02
	Disagree	4108	80.3	617	78.0	1289	82.2	2202	79.9	
	NAD	509	9.9	76	9.6	127	8.1	306	11.1	
#2: “A suicide cannot be stopped by any person, because suicide is unpreventable”.	Agree	839	16.4	165	20.9	276	17.6	398	14.4	<0.01
	Disagree	3426	67.0	500	63.2	1047	66.7	1879	68.2	
	NAD	852	16.7	126	15.9	246	15.7	480	17.4	
#3: “Something good will happen if a person chooses to live rather than die by suicide”.	Agree	4243	82.9	632	79.9	1272	81.1	2339	84.8	<0.01
	Disagree	402	7.9	71	9.0	152	9.7	179	6.5	
	NAD	472	9.2	88	11.1	145	9.2	239	8.7	
#4: “The decision whether to live or to die should be entrusted to the other individual’s personal judgment”.	Agree	1870	36.5	277	35.0	568	36.2	1025	37.2	0.21
	Disagree	2367	46.3	375	47.4	771	49.1	1221	44.3	
	NAD	880	17.2	139	17.6	230	14.7	511	18.5	
#5: “The idea of suicide pact should be abandoned when there are one or more children to consider”.	Agree	293	5.7	52	6.6	84	5.4	157	5.7	0.70
	Disagree	4457	87.1	685	86.6	1392	88.7	2380	86.3	
	NAD	367	7.2	54	6.8	93	5.9	220	8.0	
#6: “Individuals who have died by suicide must have experienced an extremely difficult time”.	Agree	3782	73.9	604	76.4	1159	73.9	2019	73.2	0.08
	Disagree	643	12.6	95	12.0	214	13.6	334	12.1	
	NAD	692	13.5	92	11.6	196	12.5	404	14.7	

*p* for trend by the Mantel Haenszel test; FAM: members of the participant’s immediate family and other relatives; FRND: friends, business associates, partners and others; None: no suicide in the participant’s life; “Agree”, “Disagree” and “NAD” represent “agree and agree a little”, “disagree and disagree a little” and “neither agree nor disagree”, respectively.

**Table 2:** Relationships of attitudes #1 and #2 toward suicide with depressive symptoms and sociodemographic characteristics, by the logistic regression analysis.

Variables	#1: When a Person Chooses to Die by Suicide, the Suicide Is Inevitable.							#2: A Suicide Cannot Be Stopped by Any Person, Because Suicide Is Unpreventable.						
	*n*	Crude Odds Ratio			Adjusted Odds Ratio			*n*	Crude Odds Ratio			Adjusted Odds Ratio		
		Odds Ratio	95% CI	*p*-Value	Odds Ratio	95% CI	*p*-Value		Odds Ratio	95% CI	*p*-Value	Odds Ratio	95% CI	*p*-Value
Sex														
Men	2166	1.94	1.61–2.35	<0.01	2.13	1.70–2.76	<0.01	2040	1.43	1.23–1.66	<0.01	1.44	1.21–1.72	<0.01
Women	2442	1.00	reference		1.00	reference		2225	1.00	reference		1.00	reference	
Age														
20–29	446	1.00	reference		1.00	reference		417	1.00	reference		1.00	reference	
30–39	771	1.36	0.88–2.11	0.17	1.31	0.81–2.10	0.27	725	1.29	0.92–1.80	0.14	1.18	0.83–1.69	0.36
40–49	844	1.42	0.92–2.19	0.11	1.32	0.83–2.10	0.25	777	1.33	0.96–1.86	0.09	1.20	0.84–1.71	0.32
50–59	966	1.65	1.09–2.50	0.02	1.56	0.99–2.46	0.07	895	1.33	0.96–1.84	0.08	1.21	0.86–1.72	0.28
60–69	1008	1.72	1.14–2.61	0.01	1.59	0.99–2.55	0.05	920	1.59	1.16–2.19	<0.01	1.46	1.02–2.09	0.04
over 70	573	2.69	1.76–4.12	<0.01	2.46	1.48–4.08	<0.01	531	2.64	1.90–3.68	<0.01	2.34	1.58–3.47	<0.01
Occupational status														
Full-time employee	1780	1.00	reference		1.00	reference		1667	1.00	reference		1.00	reference	
Part-time employee	744	1.02	0.77–1.37	0.87	1.38	1.00–1.89	0.05	682	0.87	0.68–1.10	0.26	0.97	0.74–1.25	0.79
Business owner/freelance professional	675	1.37	1.04–1.81	0.02	1.20	0.89–1.61	0.24	624	1.32	1.06–1.66	0.02	1.16	0.91–1.48	0.23
Housewife/househusband	585	0.86	0.62–1.20	0.37	1.21	0.82–1.78	0.34	525	1.11	0.87–1.43	0.41	1.19	0.89–1.61	0.23
Unemployed	557	1.51	1.13–2.01	<0.01	1.13	0.79–1.62	0.49	520	1.69	1.34–2.13	<0.01	1.22	0.93–1.64	0.14
Student	77	0.65	0.26–1.64	0.37	0.95	0.35–2.58	0.92	69	0.70	0.34–1.43	0.38	0.90	0.41–1.90	0.75
Others	190	2.43	1.65–3.57	<0.01	2.09	1.36–3.22	<0.01	178	1.93	1.36–2.73	<0.01	1.51	1.03–2.21	0.03
K6 score ≥5	1587	1.89	1.57–2.28	<0.01	2.13	1.75–2.59	<0.01	1496	1.64	1.41–1.91	<0.01	1.80	1.53–2.11	<0.01
K6 score <5	3021	1.00	reference		1.00	reference		2769	1.00	reference		1.00	reference	
Suicide in the participant’s life														
FAM	715	1.41	1.09–1.80	<0.01	1.39	1.07–1.79	0.01	665	1.56	1.27–1.92	<0.01	1.57	1.27–1.95	<0.01
FRND	1442	1.05	0.85–1.30	0.66	1.00	0.80–1.24	0.96	1323	1.25	1.05–1.48	0.01	1.25	1.05–1.49	0.01
None	2451	1.00	reference		1.00	reference		2277	1.00	reference		1.00	reference	

FAM: members of the participant’s immediate family and other relatives; FRND: friends, business associates, partners and others; None: no suicide in the participant’s life.

## Abbreviations

The following abbreviations are used in this manuscript:

K6	Kessler 6
Agree	agree and agree a little
Disagree	disagree and disagree a little
NAD	neither agree nor disagree
FAM	members of the participant’s immediate family (*i.e.*, parent, child, spouse, or sibling) and other relatives (*i.e.*, grandparent, cousin, uncle or aunt)
FRND	friends, business associates, partners and others
None	no suicide in the participant’s life
